# Molecular Characterization of TGF-Beta Gene Family in Buffalo to Identify Gene Duplication and Functional Mutations

**DOI:** 10.3390/genes13081302

**Published:** 2022-07-22

**Authors:** Muhammad Saif-ur Rehman, Faiz-ul Hassan, Zia-ur Rehman, Iqra Ishtiaq, Saif ur Rehman, Qingyou Liu

**Affiliations:** 1 Guangdong Provincial Key Laboratory of Animal Molecular Design and Precise Breeding, School of Life Science and Engineering, Foshan University, Foshan 528225, China; shsaifurrehman@yahoo.com; 2 State Key Laboratory for Conservation and Utilization of Subtropical Agro-Bioresources, Guangxi University, Nanning 530005, China; f.hassan@uaf.edu.pk; 3 Institute of Animal and Dairy Sciences, Faculty of Animal Husbandry, University of Agriculture, Faisalabad 38040, Pakistan; iqraishtiaq411@gmail.com; 4 Toba Tek Singh Campus, University of Agriculture, Faisalabad 38040, Pakistan; drzia@hotmail.com

**Keywords:** buffalo TGF-β superfamily, evolution, characterization, mutations, functional effects

## Abstract

The TGF-β superfamily is ubiquitously distributed from invertebrates to vertebrates with diverse cellular functioning such as cell adhesion, motility, proliferation, apoptosis, and differentiation. The present study aimed to characterize the TGF-β gene superfamily in buffalo through evolutionary, structural, and single nucleotide polymorphism (SNPs) analyses to find the functional effect of SNPs in selected genes. We detected 32 TGF-β genes in buffalo genome and all TGF-β proteins exhibited basic nature except INHA, INHBC, MSTN, BMP10, and GDF2, which showed acidic properties. According to aliphatic index, TGF-β proteins were thermostable but unstable in nature. Except for GDF1 and AMH, TGF-β proteins depicted hydrophilic nature. Moreover, all the detected buffalo TGF-β genes showed evolutionary conserved nature. We also identified eight segmental and one tandem duplication event *TGF-β* gene family in buffalo, and the ratio of Ka/Ks demonstrated that all the duplicated gene pairs were under selective pressure. Comparative amino acid analysis demonstrated higher variation in buffalo *TGF-β* gene family, as a total of 160 amino acid variations in all the buffalo TGF-β proteins were detected. Mutation analysis revealed that 13 mutations had an overall damaging effect that might have functional consequences on buffalo growth, folliculogenesis, or embryogenesis.

## 1. Introduction

Transforming growth factor beta (*TGF-β*) is a diverse gene family that contains a variety of growth factors, and all the members of this family have a set of three isoforms, *TGF-1*, *TGF-2*, and *TGF-3*, which are made up of interconnected dimeric polypeptide chains [1,2]. The TGF-β superfamily is ubiquitously present in both invertebrates and vertebrate species and plays a vital role in dorsoventral modeling, mesodermal initiation and patterning, and also in limb bud development, neuronal differentiation, and bone and cartilage formation [3,4,5]. The functional diversity of the *TGF-β* superfamily is crucial for the development of different body tissues and organs, particularly in vertebrates [6]. Furthermore, these proteins have a critical role in regulating and mediating basic cellular processes including cell motility, adhesion proliferation, apoptosis, and differentiation, as well as processes at the tissue or organism level, such as angiogenesis, growth, propagation, wound healing, and fibrosis [7].

The *TGF-β* superfamily members play a significant role in controlling the gene expression [8], and they can also regulate the expression of noncoding RNAs, such as microRNAs (miRNAs) and long noncoding RNAs (lncRNAs) [9]. In vertebrates, more than 30 genes belonging to the *TGF-β* superfamily have been reported so far, including *TGF-β* isoforms (*TGF-β1*, *TGF-β2*, and *TGF-β3*), bone morphogenetic proteins (*BMPs*), growth differentiation factors (*GDFs*), activins, inhibins, nodals, and Müllerian inhibitory factor (*MIF*) [7]. The *TGF* superfamily functions via Smads, which are well-defined downstream mediators. Smads control the gene expression either by activating or repressing a gene by interacting with high-affinity DNA-binding transcription factors and transcription co-regulators [8].

Members of the *TGF*-gene family have a key role in bovine physiology, for example, bone morphogenetic protein 1 (*BMP1)* contributes in the selection and dominance of follicles by regulating the proliferation and apoptosis of granulosa cells [10]. Similarly, bone morphogenetic protein/suppressor against decapentaplegic (*BMP4/SMAD*) signaling pathway has been suggested to play a role in regulation of follicular development by regulation granulosa cells in bovines [11]. Further, the bone morphogenetic protein 4 (*BMP4*) knockdown in bovines cumulus cells inhibited the proliferation of cumulus cells, apoptosis, and cell cycle arrest [12]. Growth differentiation factor-9 (*GDF9*) and bone morphogenetic protein 15 (*BMP15*) are also important candidate genes for oocyte maturation and embryo development and they were found to be highly expressed in the oocytes and embryos of buffalo [13]. Similarly, anti-Müllerian hormone (AMH) is also important for development and proper functioning of corpus luteum in buffalo [14]. The Inhibin Subunit Beta A (*INHBA*) gene was characterized in buffalo bulls and single amino acid variations detected at cleaving sites with potential association with growth, maintenance, and reproduction [15]. Moreover, myostatin (*MSTN)* is involved in skeletal muscle growth, and additionally was also found to be involved in folliculogenesis [16]. Water buffalo is an economically important genetic asset that contributes more than 15% of the world’s total milk supply [17]. Despite having huge commercial importance, buffaloes are being neglected for their genetic resources, especially the breeding- and physiology-related regulators. No comparative genomic studies are available on buffalo and cattle to investigate poor estrus expression and lower reproductive efficiency in buffalo compared to cattle. Therefore, there is a dire need to shed light on the biological entities that would help to develop our understanding with different regulatory gene families which ultimately benefit buffalo development. The present study aimed to explore the evolutionary, physicochemical characterization, and gene structure analyses of the *TGF-β* superfamily in buffalo. Further, we also conducted a single nucleotide polymorphism (SNPs) analysis to find the functional effect of SNPs in selected genes of the *TGF-β* superfamily in buffalo.

## 2. Materials and Methods

### 2.1. Identification of TGF-β Genes in Buffalo

Whole-genome, proteome, and annotation data of Mediterranean river buffalo (UOA_WB_1), cattle (ARS-UCD1.2), sheep (Oar_rambouillet_v1.0), goat (ARS1), human (GRCh38.p12), and horse (EquCab3.0) were downloaded from National Center for Biotechnology Information (NCBI) Genome database (https://www.ncbi.nlm.nih.gov/) (accessed on 2 March 2022). Both the Basic Local Alignment Search Tool (BLAST) and hidden Markov model (HMM) searches were performed to identify all TGF-β protein isoforms in buffalo at the genome level. The non-redundant *TGF-β* gene sequences of cattle (*Bos taurus*), human (*Homo sapiens*), goat (*Capra hircus*), horse (*Equus caballus*), and sheep (*Ovis aries*), were retrieved from the UniProt (https://www.uniprot.org/) (accessed on 2 March 2022), and subjected as a query via BLASTp with a threshold of e-value = 10^−5^ by using BLOSUM62 matrix with a six word size, eleven gap cost with an extension of 1, and a conditional composition score matrix adjustment. Additionally, the buffalo dataset was also searched with HMMER [18,19] (http://hmmer.org/) (accessed on 3 March 2022) software using HMM profile of the TGF-β domain (PF00019) from the Pfam online database [20] with an E-value 1.0 × e^−5^. To avoid ambiguity, duplicate sequences were deleted after retrieving the relevant protein sequences. To confirm the TGF-β domains in protein sequences, these non-redundant sequences were analyzed in Simple Modular Architecture Research Tool (SMART) (http://smart.embl-heidelberg.de/) (accessed on 4 March 2022), and NCBI-CDD database was used for buffalo TGF-β proteins conserved domains searches.

### 2.2. Characterization of Buffalo TGF-β Genes

The ProtParam tool (https://web.expasy.org/protparam/) (accessed on 7 March 2022) was used to analyze the physiochemical features of buffalo TGF-β proteins, including the number of amino acids (A.A), instability index (II), molecular weight (MW), isoelectric point (pI), aliphatic index (AI), and grand average of hydropathicity (GRAVY) [21].

### 2.3. Multiple Sequence Alignment

To identify the indels and visualize sequence variations, all the TGF-β protein sequences were aligned using Multiple Align Show (https://www.bioinformatics.org/sms/multi_align.html) (accessed on 7 March 2022).

### 2.4. Structural Features Analysis

The conserved motifs were evaluated in the MEME suite (https://meme-suite.org/meme/tools/meme) (accessed on 8 March 2022) [22]. All the buffalo TGF-β protein sequences were given in *FASTA format* as query. Site distribution was selected as one occurrence per sequence to find 10 MEME motifs with the minimum and maximum motif widths ranging between 6 and 50, respectively. To examine the pattern of introns and exons in *TGF-β* genes, CDs and genomic sequences were loaded in Gene Structure Display Server (GSDS) (http://gsds.gao-lab.org/) (accessed on 10 March 2022) and then the Tbtools (v1.098721) software (https://github.com/CJ-Chen/TBtools) (accessed on 10 March 2022), which used in-house scripts general feature format (GFF) file to depict the final gene structure [23].

### 2.5. Phylogenetic Analysis

All the TGF-β amino acid sequences of cattle, buffalo, goat, sheep, horse, and human were aligned in ClustalW, and the neighbor-joining (NJ) molecular phylogenetic tree using MEGA7 v.7.0 software (https://megasoftware.net/) (accessed on 10 March 2022) was constructed with a bootstrap value of 1000 replicates, adopting the Poisson model with pairwise deletion [24].

### 2.6. Synteny and Gene Duplications Analysis of TGF-β Superfamily Genes

Chromosomal locations of buffalo *TGF-β* genes were acquired from their genome resources, and a genome annotation (GFF) file was given as an input to the MCScanX program to map the physical locations of genes on chromosomes and then visualized in TBtools. Furthermore, the buffalo and cattle dual synteny plots were aligned for *TGF-β* genes collinearity [25]. Additionally, pairwise alignment of homologous gene pairs of *TGF-β* genes using MEGA7 v.7.0 with the MUSCLE algorithm was used to assess the occurrences of duplications for the buffalo TGF-β gene family [24]. DnaSP v6.0 software (http://www.ub.edu/dnasp/) (accessed on 10 March 2022) was also used to estimate pairwise synonymous substitutions per synonymous site (Ks) and nonsynonymous substitutions per nonsynonymous site (Ka) adjusted for multi hits [26].

### 2.7. Evaluation of Functional Mutation (SNPs) Effect in Buffalo TGF-β Proteins

The TGF-β proteins amino acid sequences of buffalo and cow were aligned using ClustalW, and the mutations were visualized using BioEdit software (v7.2) (https://bioedit.software.informer.com/7.2/, accessed on 12 March 2022). To check the impact of these variations/mutations between buffalo and cow, different online tools were used, including Sequence homology-based methods SIFT (Sorting Intolerant from Tolerant) (http://blocks.fhcrc.org/sift/SIFT.html, accessed on 12 March 2022), Provean (http://provean.jcvi.org/, accessed on 12 March 2022), Protein sequence and structure-based methods (PolyPhen 2; Polymorphism Phenotyping v2, accessed on 12 March 2022), Predictor of effects of single point protein mutation (I-Mutant; http://gpcr2.biocomp.unibo.it/cgi/predictors/I-Mutant3.0/I-Mutant3.0.cgi, accessed on 12 March 2022), phdSNP (https://snps.biofold.org/phd-snp/phd-snp.html, accessed on 12 March 2022), and Prediction of Protein Stability Changes for Single Site Mutations from Sequences (Mupro (http://mupro.proteomics.ics.uci.edu/, accessed on 12 March 2022).

## 3. Results

### 3.1. Genomic Identification of Buffalo TGF-β Genes

**Figure 1:**
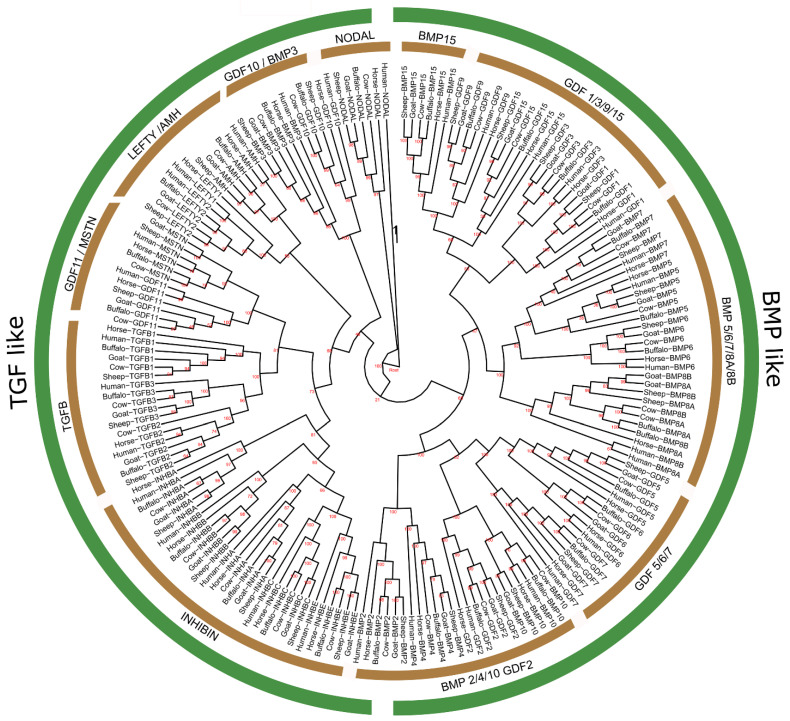
Phylogenetic tree analysis of TGF-β gene family in six representative species.

By using the BLAST and HMMER software, 193 non-redundant protein sequences encoded by 32 *TGF-β* genes were detected from the whole genomes of five mammalian species (cattle, buffalo, sheep, goat, and horse) while the human sequences were encoded by 33 *TGF-β* genes. Similarly, about 32 genes of the *TGF-β* gene family were also identified from the buffalo genome (Figure 1).

### 3.2. Phylogenetic Analysis of TGF-β Gene Family

Phylogenetic analysis of *TGF-β* genes of six mammalian species was executed and all those identified genes were clustered into two major clades, the *TGF-β*-like and *BMP*-like, where the TGF-β-like clade was further categorized into six sub-groups, such as *NODAL*, *GDF10/BMP3, LEFTY/AMH, GDF11/MSTN, TGF β*, and *INHIBIN*, while *BMP*-like has only five, including *BMP2/4/6/, GDF2, GDF5/6/7, BMP5/6/7/8A/8B, GDF1/3/9/15*, and *BMP15* (Figure 1). All the genes of the *Bubalus bubalis TGF-β* gene family shared higher sequence homology with *Bos taurus*, as compared to *Capra hircus* and *Ovis aries*. The generated dendrogram (Figure 1) also revealed that the *TGF-β* gene family shows a close evolutionary relationship with other representative mammals.

### 3.3. Gene Structure Analysis of Buffalo TGF-β Genes

**Figure 2:**
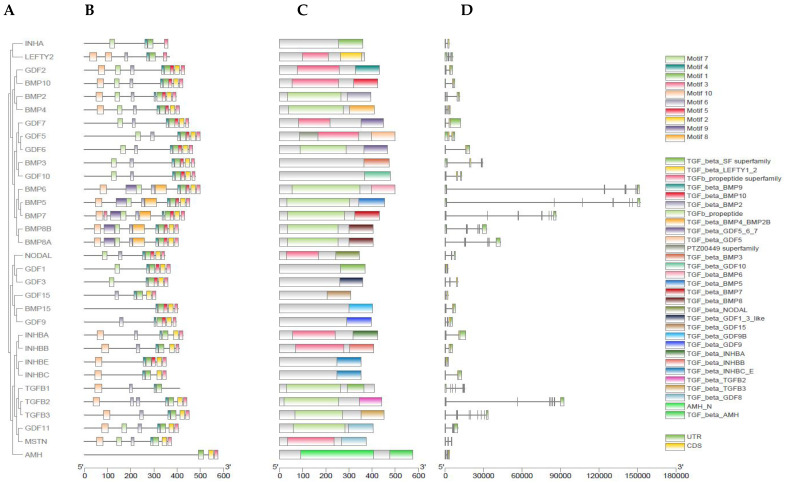
(**A**) Phylogenetic relationship of buffalo 32 TGF-β genes. (**B**) Motif patterns. (**C**) Conserved domain regions. (**D**) Gene structure.

The phylogenetic relationship, motif distribution, gene structure, and conserved region analyses were conducted to discover the structural features of *TGF-β* gene family in buffalo, as shown in Figure 2A–D. These analyses were executed while taking their phylogenetic evolutionary relationships (Figure 2A). From buffalo *TGF-β* genes, a total of 10 MEME conserved motifs were identified, of which motifs MEME-8 and MEME-9 have a higher number of amino acids, 49 and 45, respectively, and both the motifs were annotated as TGF-β domain after the Pfams search (Figure 2B and Table 1). Additionally, the results were also checked against the NCBI-CDD database for verification (Figure 2C). Additionally, along with the domain of TGF beta, the domain of AMH-N and PTZ00449 superfamily were also identified. Moreover, the gene structure analysis showed that the introns and the upstream and downstream untranslated regions (UTRs) structure varied greatly and all the buffalo *TGF-β* genes had different numbers of exon and intron patterns (Figure 2D).

### 3.4. Characterization of Physicochemical Properties of Buffalo TGF-β Genes

Physiochemical attributes of the buffalo *TGF-β* gene family, including *TGF*-like and *BMP*-like, were analyzed for their chromosomal distribution, exon count, number of the amino acids (A.A) in each peptide, molecular weight (Da), isoelectric point (pI), instability index (II), aliphatic index (AI), and Grand Average of hydropathicity Index (GRAVY), as presented in Table 2 and Table 3. The molecular weight of buffalo TGF-β proteins ranged from 34,638.86 to 55,133.38 Da and the values of pI from 4.97 to 10.42. All the proteins show a basic nature except for *INHA*, *INHBC*, *MSTN*, *BMP10*, and *GDF2,* which show acidic properties (Table 2 and Table 3), and the aliphatic index values were found to be >65, which exhibited thermostable characteristics of all the buffalo TGF-β proteins. According to the instability index, all the proteins appeared unstable as values were >40 (Table 2 and Table 3). Owing to lower GRAVY values, all the buffalo TGF-β proteins had hydrophilic nature except for *GDF1* and *AMH,* which depicted hydrophobic nature (Table 2 and Table 3).

### 3.5. Buffalo and Cattle TGF-β Genes Collinearity Analysis and Chromosomal Distribution

**Figure 3:**
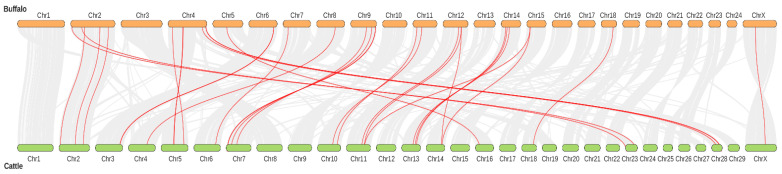
Buffalo and cattle *TGF-β* genes collinearity analysis. The chromosomes of the cattle are presented in green, and buffalo in yellow color.

All the buffalo *TGF-β* genes were located on 13 chromosomes, whereas all these *TGF-β* genes were randomly present on 15 chromosomes in cattle. Moreover, the majority of the buffalo *TGF-β* genes were mainly positioned on the distal ends of the chromosomes (Figure 3). The duplication events of the buffalo TGF-β gene family were studied to better understand evolutionary history (Table 4). Nine duplicated pairs of genes were detected, of which eight homologous gene pairs including *TGFB1/TGFB3*, *TGFB2/TGFB3*, *BMP4/BMP2*, *BMP3/GDF10*, *BMP7/BMP5*, *BMP8A/BMP7*, *GDF9/BMP15*, and *GDF10/BMP1* were presumed as segmental duplications, while the *BMP6/BMP5* was the only homologous gene pair supposed to be in tandem duplication (Table 4 and Figure S1). Further, the ratio of nonsynonymous substitutions per nonsynonymous site (Ka) to synonymous substitutions per synonymous site (Ks) were calculated for these homologous gene pairs, and the four gene pairs (*TGFB1/TGFB3*, *TGFB2/TGFB3*, *BMP3/GDF10*, and *BMP8A/BMP7*) had Ka/Ks ratios > 1, whereas five gene pairs (*BMP4/BMP2*, *BMP7/BMP5*, *BMP6/BMP5*, *GDF9/BMP15*, and *GDF10/BMP15*) showed <1 Ka/Ks ratio (Table 4).

### 3.6. Comparative Amino Acid and Functional Mutation Effect Analysis of Buffalo TGF-β Gene Family

Comparative amino acid analysis for all the *TGF-β* genes of buffalo was conducted using cattle as a reference (Figures S2–S33). Four buffalo *TGF-β* genes, including *TGFB2*, *TGFB3*, *INHBA*, and *BMP7,* shared 100% homology with cattle genes (Figures S2–S5), while the indels were also observed in buffalo and cattle *INHBE*, *GDF6*, *BMP6*, *TGFB1,* and *GDF11* genes, where four indels in each of *INHBE* and *GDF6*, three in *BMP6,* and one in each of *TGFB1* and *GDF11* were assessed (Figures S6–S10). Furthermore, a total of 160 amino acid alterations were calculated in all the buffalo *TGF-β* genes, where a single amino acid variation was detected in each *BMP2*, *BMP4*, and *BMP10* gene (Table 5). Similarly, *TGFB1*, *INHBB*, *BMP5*, *GDF1*, *GDF5,* and *GDF11* had two amino acid changes but two genes (*GDF6* and *GDF15*) and four genes (*MSTN*, *BMP6*, *BMP8B,* and *GDF3*) with three and four amino acids differences, respectively, were also found (Table 5). Moreover, *GDF7* with six, *INHBC*, *INHBE*, *NODAL*, and *GDF10* with seven, only *BMP3* with eight, *INHA*, *GDF2*, and *GDF9* with nine, and *BMP8A* and *BMP15* with ten amino acid substitutions were also evaluated in buffalo (Table 5). In addition, higher ratio of non-synonymous nucleotide substitution was perceived in *LEFTY2* and *AMH* genes, with 16 and 18 amino acids replacements in buffalo (Table 5 and Figures S6–S33). Additionally, the functional effect of these mutations was assessed using different software (Table 5) and a total of 13 detected amino acid mutations in different *TGF-β* genes of buffalo, including *MSTN* (E116 > D), *BMP3* (R287 > W), *BMP6* (Y419 > C), *BMP8A* (A145 > V & R305 > G), *BMP8B* (R305 > G), *BMP15* (G272 > R & E384 > Q), *GDF1* (S9 > G), *GDF9* (L49 > F & P77 > S), *GDF11* (A40 > G), and *AMH* (A334 > T), due to nucleotides non-synonymous alterations were supposed to have overall damaging effect on protein structure and functions, while the other amino acid substitutions have overall neutral affect and caused no serious influence on protein structure and functions (Table 5).

## 4. Discussion

The advancements in high-throughput genome sequencing technologies, typically the next-generation sequence data availability, make it easy to scan the genetic variability of genes, such as SNPs ordering with their functional effect which control a specific phenotypic trait that allow to understand animals’ genetics at molecular level [27,28,29,30,31,32]. For farm animals, the candidate gene studies analyze available genetic resources to identify functional genes and their potential association with productivity traits, such as disease resistance, production ability, and adaptation [29]. In buffaloes, comparative genomics offers an opportunity to explore the genetics of economically important physiological traits though discovering novel genes and their regulatory mechanisms, which significantly contributes to the development of the buffalo industry [32].

The *TGF*-gene family has a set of coding genes with conserved structure and biologically diverse functioning that release signaling molecules which regulate fundamental cellular pathways, and dynamic biological processes such as apoptosis, communication, proliferation, differentiation, and tissue remodeling throughout growth, repair, and organogenesis [33,34]. In the present study, 32 *TGF-β* coding genes were identified from buffalo genome that were categorized into two major groups (*TGF-β*-like and *BMP*-like), where the *TGF-β*-like had six while *BMP*-like had only five sub-groups or set of genes sharing higher sequence homology with *Bos taurus* than *Capra hircus* and *Ovis aries* (Figure 1). The phylogenetic pattern observed in our study is in agreement with earlier findings reporting the evolution of the *TGF*-gene family in 9 invertebrates and 15 chordates species [35]. Furthermore, TGF-β peptide was the only conserved motif found in the buffalo *TGF*-gene family (Table 1).

All the members of the *TGF-β* superfamily are predominantly involved in a variety of cellular activities by binding to a particular receptor to generate a signal for a specific cellular function. Except for inhibin and *GDNF* subfamilies, all the other *TGF*-superfamily ligands bind to a unique set of double transmembrane Ser/Thr kinase receptors, type I and type II receptors [36]. Firstly, the dimeric ligand binds to cognate type II receptors and then this complex recruits or activates type I receptors, eventually causing R-SMAD phosphorylation and activation (receptor-regulated SMADs) [37]. Generally, there are two models for R-SMAD activation, firstly, SMAD1/5/8 interact with *AMH*, *BMPs,* and some *GDFs* through ALK-2, -3, and -6; secondly, SMAD2/3 respond to activins, *NODAL TGF-βs*, and some *GDFs* via ALK-4, -5, and -7. Finally, the activated R-SMADs form a complex with SMAD (Co-SMAD) or SMAD4, and then this complex could translocate into the nucleus and influence or regulate the target gene expression through interacting with other transcription factors in different cell types or tissues [37,38].

The *GDF1* and *GDF3* could interact with nodal and initiated signals for the long term, which are important in left–right patterning [39], while *GDF3* is substantially involved in robust nodal signaling during germ layer formation [40]. Further, *AMH* which is distributed in Sertoli cell of testes, have functional involvement in gonads development and sex differentiation through inhibiting the Müllerian duct by smad1 pathway [41]. Moreover, the *GDF8* (myostatin) and *GDF11* act through type II receptors (ActRII or ActRIIB) or type I receptors (ALK-4 or -5 type) that activate the Smad2/3 pathway and regulate the muscle mass by inhibiting the muscle differentiation and regeneration [42,43,44,45]. It is unlikely that GDF5 is involved in muscle hypertrophy through BMP signaling [46]. The *BMP9* and *BMP10* are recognized as ligands of ALK-1 receptor which crucially regulate vascular system homeostasis and heart development [47,48,49], while BMP6 controls the iron metabolism and expression of hepcidin by acting as an endogenous ligand [50,51].

Similarly, the *GDF9* and *BMP15* are functionally expressed in oocytes and work synergistically through the smad3 pathway via ERK1/2 and SRC kinase-dependent signaling [52]. Although *GDF10* and *BMP3* are closely related and have positive role in endochondral bone formation in adult animals, they negatively affect the bone morphogenesis at embryonic stage [53]. Additionally, BMP8A is involved in the regulation of spermatogenesis through smad2/3 and smad1/5/8 pathways and BMP8B prevents the male germ cells apoptosis [54,55], whereas *GDF5/6/7* contributes to the formation of normal bones, limb joints, skull, and axial skeleton [56].

Organisms use gene duplication mechanisms including retroposition, genome, or chromosomal duplication, and crossing over to acquire novel gene or genetic variants, which tremendously contributes to the evolution of functional processes [57]. Discovering the dynamics that create duplicate gene copies, as well as the subsequent trajectories among duplicated genes, is critical because these studies shed light on localized and genome-wide attributes of evolutionary factors that influence intra- and inter-specific genome contents, evolutionary interactions, and relationships [57]. It is difficult to measure the rate of duplications that occur but selective pressure and mutations with functional effects are vital for developing redundant genetic variants [58]. Over generations of an organism, the duplicated gene could accumulate mutations faster than in a single functional gene copy and possibly develop a novel function [59]. Earlier, it has been reported that in ice fish, the apparent mutations in a duplicated digestive gene transform to the antifreeze gene while duplication leads to a distinct snake venom gene [59], and in pigs, synthesis of 1-β-hydroxytestosterone [60]. In the present study, we identified eight segmental and one tandem duplication event in the buffalo *TGF-β* gene family. The Ka/Ks ratio for duplicated gene pairs demonstrated that all these gene pairs were under selective pressure, among which four gene pairs were under positive selection with Ka/Ks ratio > 1, while five gene pairs had Ka/Ks ratio < 1, exhibiting that these are purifying selection pressure [61]. Our findings are consistent with earlier studies and suggest that observed gene duplication in the *TGF-β* gene family resulted in an increased buffalo genome size and complexity [62].

In mammals, seven receptors belonging to type I called activin receptor (kinase-like 1–7) and five type II receptors (such as ACVR2A, ACVR2B, AMHR2, BMPR2, and TβR2) have already been identified that can induce the heterotetrameric complex [38]. In this reverence, the sequence and structural variations in *TGF-β* superfamily ligands should be crucial for their differential binding affinities for distinct type I and type II receptors. Furthermore, the type II receptor could also influence the binding affinity of ligands with type I receptors [63]. The comparative amino acid analysis of our study presented a total of 160 amino acid substitutions in buffalo *TGF-β* genes, of which 13 exhibited an overall damaging effect on respective protein structure and functions. Except for *GDF3*, *GDF9*, and *BMP15*, all *TGF-β* superfamily ligands use an extra conserved cysteine for intermolecular disulphide bond formation, which stabilizes the dimer [64]. Even though the majority of ligands tend to be homodimers, ligand heterodimerization has also been reported [64], such as heterodimerization between *GDF9* and *BMP15* [65], *GDF1* and *NODAL* [39], and activin βA–βB [64].

Likewise, the members of the *TGF-β* superfamily play a dynamic role in controlling different biological processes such as folliculogenesis [66], skeleton development [67], nodal signaling [40], and fat yield [68], while absence or altered expression of these genes can induce impaired development. For example, *GDF3* is important for folliculogenesis [66], skeleton development [67], and nodal signaling [40], but zebrafish mutant embryos showed early embryonic lethality [40]. Similarly, in humans it has been reported that mutations in *GDF3* cause ophthalmic and skeletal abnormalities, and the patients with ocular and/or skeletal (Klippel–Feil) anomalies were found to have several missense variations, including one with heterozygous changes in *GDF3* and *GDF6* [69]. Additionally, *GDF6,* which is important for proper development of bones and joints in the limbs, head, and axial skeleton but abnormal development of joints, ligaments, and cartilage, could occur in the absence of the *GDF6* gene [56], and *GDF6* mutations in zebrafish have been reported associated with reduced eye size and different skeletal defects [70].

On the other hand, *GDF9* is primarily involved in folliculogenesis and controls the development of theca and granulosa cells, as well as plays an important role in oocyte differentiation and maturation [71,72]. In sheep, *GDF9* natural mutations resulted in higher ovulation rate and twin or triplet births in heterozygotes, while in homozygotes a complete primary ovarian failure leading to complete sterility was also reported [73]. Furthermore, the polymorphism in the *GDF9* gene in sheep was also associated with litter size, milk production, and prolificacy [74,75]. In buffalo, *GDF9* plays a significant role in buffalo oogenesis as it is present throughout folliculogenesis and embryogenesis stages, while during follicular development, the *GDF9* gene surged at first, then reduced [76]. Moreover, alterations in the maternal mRNA transcript of *GDF9* gene in buffalo oocytes resulted in the deleterious seasonal effects on oocytes development [77]. In the present study, we identified a total of nine amino acid substitutions, of which one belongs to *GDF3*, three to *GDF6* along with four amino acid deletion, and five associated with *GDF9*. Further, the functional mutation analysis revealed that only two mutations of *GDF9* exhibited damaging effect, which might have functional influence on folliculogenesis or embryogenesis of buffalo. The present study provides evolutionary insights into the *TGF-β* gene superfamily in buffalo, revealing the functional importance of SNPs in different genes with developmental and physiological consequences on buffalo performance. The mutations identified in the present study provide a basis for further studies to investigate their potential utility for genomic selection for targeted improved breeding and utilization of buffalo genetic resources.

## 5. Conclusions

In the present study, we comprehensively described the molecular structure and functional role of mutations in the *TGF-β* superfamily in buffalo. All the *TGF-β* genes in buffalo showed evolutionary conserved nature. Eight segmental and one tandem duplication events in the buffalo *TGF-β* gene family were identified, and all the duplicated gene pairs were under selective pressure. These duplications might have played a role in adaptation and speciation to specific functions and ecological niches [78]. We identified 13 mutations in different *TGF-β* genes with an overall damaging effect that might have functional consequences on buffalo growth and development, folliculogenesis, and/or embryogenesis. The present study is the first report on comparative genome-wide characterization of the *TGF-β* superfamily in buffalo and it provides insight into the evolutionary importance of gene duplications and mutations in the *TGF-β* superfamily with respect to adaptation and speciation. Furthermore, these findings would be helpful in understanding the crucial role of these genes and their potential utility for selective breeding in buffalo for economically important traits such as reproduction, growth, and development. Further studies are warranted to elucidate functional effects of mutations identified in different *TGF-β* genes in the present study to confirm their physiological manifestation in buffalo and potential effects on growth, development, and reproductive performance in buffalo.

## Figures and Tables

**Table 1:** Ten substantially conserved motifs identified in *TGF-β* gene family of buffalo.

MEME Motif	Amino Acid Sequence	Length	Pfam Domain
1	WIIAPKGYEANYCEGECPFPLASH	24	-
2	VPKPCCVPTKLSPJSILYFDD	21	-
3	LKKYPBMVVEECGCR	15	-
4	LYVDFQDLGW	10	-
5	LNPTNHAIIQTLVHL	15	-
6	SGWLVFDVTAAVRRW	15	-
7	FBLSSIPDGEAVTAAELRJYK	21	-
8	SPQKBLGLQLYVETDDGRSIBPGLAGLVGRQGPRSKQPFMVAFFKASEV	49	TGFb_propeptide
9	AGDPPLASGQDERFLGDADMVMSFVNLVERDKEFGHQEPHHKEFR	45	TGFb_propeptide
10	LEAIKRZILSKLGLPSRPRPSRPPPKPPL	29	-

**Table 2:** Physicochemical properties of the TGF-like genes of the buffalo *TGF-β* gene family.

Gene	Chr.	Exon count	A.A.	MW (Da)	pI	II	Al	GRAVY
*TGFB1*	18	8	410	46,776.39	7.98	53.22	80.88	−0.463
*TGFB2*	5	8	442	50,550.99	8.74	53.80	80.07	−0.406
*TGFB3*	11	8	452	51,418.09	8.28	50.18	86.26	−0.392
*INHA*	2	2	360	38,828.70	6.91	65.11	85.42	−0.093
*INHBA*	8	2	425	47,521.45	8.10	63.72	78.47	−0.497
*INHBB*	2	2	408	45,056.70	8.72	56.03	80.27	−0.262
*INHBC*	4	2	352	38,480.15	6.59	46.01	88.89	−0.045
*INHBE*	4	2	352	38,731.98	9.95	57.93	91.45	−0.171
*NODAL*	4	3	346	39,932.99	8.07	60.21	84.83	−0.360
*MSTN*	2	3	375	42,495.89	6.05	41.03	85.79	−0.332
*BMP3*	7	3	475	53,458.10	9.04	66.66	78.44	−0.556
*GDF10*	4	3	478	52,683.43	9.55	55.40	76.63	−0.450
*GDF11*	4	3	355	37,982.55	8.28	59.40	78.30	−0.428
*AMH*	9	5	575	60,812.20	8.63	54.46	96.50	−0.044
*LEFTY2*	5	4	367	41,460.84	8.91	52.23	92.18	−0.251

Note: Chr. (Chromosome).

**Table 3:** Physicochemical properties of the BMP-like genes of the buffalo *TGF-β* gene family.

Gene	Chr.	Exon count	A.A.	MW (Da)	pI	II	Al	GRAVY
*BMP2*	14	4	395	44,569.82	8.96	55.51	79.75	−0.422
*BMP4*	11	6	409	46,639.95	8.57	59.49	80.54	−0.543
*BMP5*	2	7	454	51,323.27	9.10	52.12	79.49	−0.414
*BMP6*	2	7	499	54,621.73	7.96	55.48	71.24	−0.413
*BMP7*	14	7	431	49,288.88	7.36	53.76	76.96	−0.410
*BMP8A*	6	7	404	44,786.52	9.12	66.86	86.71	−0.288
*BMP8B*	6	7	405	44,683.35	8.91	65.49	85.78	−0.285
*BMP10*	12	2	424	48,424.90	4.97	49.13	84.60	−0.428
*BMP15*	X	2	403	46,096.52	9.67	48.27	93.08	−0.292
*GDF1*	9	2	369	39,252.61	9.51	71.00	93.44	0.002
*GDF2*	4	2	431	48,303.95	6.21	54.20	77.77	−0.424
*GDF3*	4	3	360	40,354.43	7.58	62.16	88.83	−0.214
*GDF5*	14	2	499	55,133.38	9.80	45.22	72.99	−0.586
*GDF6*	15	2	466	51,852.09	9.48	66.01	69.55	−0.604
*GDF7*	12	2	450	46,750.93	9.62	53.66	81.09	−0.133
*GDF9*	9	3	396	45,364.01	9.27	56.71	73.13	−0.480
*GDF15*	9	2	308	34,638.86	10.42	64.60	84.94	−0.472

**Table 4:** Analysis of the Ka/Ks ratio for each duplicated gene pair of the buffalo *TGF-β* gene family.

Gene Pairs	Chromosome	Duplication	Ka	Ks	Ka/Ks
*TGFB1/TGFB3*	18/10	SD	0.6711	0.6071	1.1
*TGFB2/TGFB3*	16/10	SD	0.5789	0.3323	1.7
*BMP4/BMP2*	10/13	SD	0.4284	0.8102	0.5
*BMP3/GDF10*	6/28	SD	0.7044	0.6483	1.08
*BMP7/BMP5*	13/23	SD	0.4044	0.6352	0.64
*BMP6/BMP5*	23/23	TD	0.5073	0.5751	0.88
*BMP8A/BMP7*	3/13	SD	0.5001	0.4318	1.15
*GDF9/BMP15*	7/X	SD	0.7914	1.0686	0.74
*GDF10/BMP15*	28/X	SD	0.8583	1.0661	0.8

SD (segmental duplication); TD (tandem duplication); Ka (non-synonymous substitutions); Ks (synonymous substitutions).

**Table 5:** Functional effects of mutations in different TGF-β gene family members.

	Mutations	Polyphen2	Mupro	Provean	I-Mutant	Phd-Snp	SNAP^2^	Predict SNP	Meta SNP	SIFT	Overall Effect
***TGFB1***											
1	D154 > E	BE	IN	NE	DE	NE	NE	NE	NE	TOL	SY
2	V155 > L	BE	IN	NE	DE	NE	NE	NE	NE	TOL	SY
***INHA***											
1	G15 > R	BE	DE	NE	IN	NE	EFF	NE	NE	NT	SY
2	L21 > P	BE	DE	NE	DE	NE	NE	NE	NE	TOL	SY
3	L23 > V	BE	DE	NE	DE	NE	NE	NE	NE	NT	SY
4	H58 > P	BE	IN	NE	IN	NE	NE	NE	NE	TOL	SY
5	T136 > I	BE	IN	NE	IN	NE	NE	NE	NE	NT	SY
6	M157 > T	BE	DE	NE	IN	NE	NE	NE	NE	NT	SY
7	P293 > T	BE	DE	NE	DE	NE	NE	NE	NE	TOL	SY
8	P300 > S	BE	DE	NE	DE	NE	EFF	NE	NE	TOL	SY
9	V309 > I	BE	DE	NE	DE	NE	NE	NE	NE	TOL	SY
***INHBB***											
1	S21 > W	BE	DE	NE	DE	NE	NE	NE	NE	NT	SY
2	S255 > G	BE	DE	NE	DE	NE	NE	NE	NE	NT	SY
***INHBC***											
1	H77 > Q	BE	IN	NE	DE	NE	NE	NE	NE	TOL	SY
2	E103 > Q	BE	DE	NE	DE	NE	NE	NE	NE	TOL	SY
3	T175 > S	BE	DE	NE	DE	NE	NE	NE	NE	TOL	SY
4	E203 > G	BE	DE	NE	DE	NE	NE	NE	NE	TOL	SY
5	R214 > G	BE	DE	NE	DE	NE	NE	NE	NE	TOL	SY
6	V221 > M	PD	DE	NE	DE	NE	NE	NE	NE	NT	SY
7	T310 > A	BE	IN	NE	DE	NE	NE	NE	NE	TOL	SY
***INHBE***											
1	L4 > P	UNK	DE	NE	IN	NE	NE	NE	NE	TOL	SY
2	T33 > A	BE	DE	NE	DE	NE	NE	NE	NE	TOL	SY
3	Q130 > H	BE	DE	NE	DE	NE	NE	NE	NE	TOL	SY
4	P195 > L	BE	DE	NE	DE	NE	NE	NE	NE	TOL	SY
5	T203 > A	BE	DE	NE	DE	NE	NE	NE	NE	TOL	SY
6	A210 > S	BE	DE	NE	DE	NE	NE	NE	NE	TOL	SY
7	R222 > Q	BE	DE	NE	DE	DIS	EFF	NE	NE	NT	SY
***NODAL***											
1	Q4 > H	BE	DE	NE	DE	NE	NE	NE	NE	TOL	SY
2	C5 > R	PD	DE	NE	IN	NE	NE	NE	NE	TOL	SY
3	T172 > M	BE	IN	NE	DE	NE	NE	NE	NE	TOL	SY
4	S173 > P	PD	IN	NE	IN	DIS	NE	NE	DIS	TOL	SY
5	S174 > T	BE	IN	NE	DE	NE	NE	NE	NE	TOL	SY
6	R182 > Q	BE	DE	NE	DE	NE	NE	NE	NE	TOL	SY
7	S185 > T	BE	DE	NE	DE	NE	NE	NE	NE	TOL	SY
***MSTN***											
1	E116 > D	PD	DE	NE	DE	NE	EFF	DEL	NE	TOL	NSY
2	T117 > A	PD	DE	NE	DE	NE	NE	NE	NE	TOL	SY
3	K141 > Q	BE	DE	NE	DE	NE	NE	NE	NE	TOL	SY
4	H275 > R	BE	DE	NE	DE	NE	NE	NE	NE	TOL	SY
***BMP2***											
1	V16 > I	BE	DE	NE	DE	NE	NE	NE	NE	TOL	SY
***BMP3***											
1	E82 > D	PD	IN	NE	DE	NE	EFF	NE	NE	TOL	SY
2	P86 > Q	BE	IN	NE	DE	NE	NE	NE	NE	TOL	SY
3	P92 > L	BE	DE	NE	DE	NE	NE	NE	NE	TOL	SY
4	K233 > T	PD	DE	NE	DE	NE	NE	NE	NE	TOL	SY
5	Q278 > H	BE	DE	NE	DE	NE	NE	NE	NE	TOL	SY
6	S281 > A	BE	DE	NE	DE	NE	NE	NE	NE	TOL	SY
7	R287 > W	UNK	DE	DEL	DE	NE	EFF	DIS	NE	NT	NSY
8	E316 > G	BE	DE	NE	DE	NE	NE	NE	NE	TOL	SY
***BPM4***											
1	D173 > E	BE	DE	NE	IN	NE	NE	NE	NE	TOL	SY
***BMP5***											
1	T25 > A	BE	DE	NE	DE	NE	NE	NE	NE	TOL	SY
2	M338 > V	BE	DE	NE	DE	NE	NE	NE	NE	TOL	SY
***BMP6***											
1	G43 > S	BE	DE	NE	DE	NE	NE	NE	DIS	TOL	SY
2	D151 > G	BE	DE	NE	DE	NE	NE	NE	NE	TOL	SY
3	S160 > P	BE	IN	NE	IN	NE	NE	NE	NE	TOL	SY
4	Y419 > C	PD	DE	DEL	DE	DIS	EFF	DEL	DIS	NT	NSY
***BMP8A***											
1	I23 > V	UNK	DE	NE	DE	NE	NE	NE	NE	TOL	SY
2	G57 > R	PD	IN	DEL	DE	NE	NE	NE	NE	TOL	SY
3	D87 > G	BE	DE	NE	DE	NE	NE	NE	NE	TOL	SY
4	D116 > N	BE	IN	DEL	DE	NE	NE	NE	NE	NT	SY
5	A145 > V	PD	IN	DEL	IN	DIS	EFF	DEL	DIS	NT	NSY
6	G258 > R	UNK	IN	NE	DE	DIS	NE	NE	NE	TOL	SY
7	P284 > A	BE	DE	NE	DE	NE	NE	NE	NE	TOL	SY
8	N294 > D	BE	DE	NE	DE	NE	NE	NE	NE	TOL	SY
9	R305 > G	PD	DE	DEL	DE	DIS	EFF	DEL	DIS	NT	NSY
10	V375 > L	BE	IN	NE	DE	NE	NE	NE	NE	TOL	SY
***BMP8B***											
1	D87 > G	BE	DE	NE	DE	NE	NE	NE	NE	TOL	SY
2	D116 > N	BE	IN	DEL	DE	DIS	NE	NE	NE	NT	SY
3	G258 > A	UNK	IN	NE	DE	NE	NE	NE	NE	TOL	SY
4	R305 > G	PD	DE	DEL	DE	DIS	EFF	DEL	DIS	NT	NSY
***BMP10***											
1	E229 > K	BE	DE	NE	DE	NE	NE	NE	NE	TOL	SY
***BMP15***											
1	V16 > A	UNK	DE	NE	DE	NE	NE	NE	NE	NT	SY
2	Q56 > L	PD	IN	NE	DE	NE	NE	NE	NE	TOL	SY
3	I62 > V	BE	DE	NE	DE	NE	NE	NE	NE	TOL	SY
4	Q75 > H	BE	DE	NE	DE	NE	NE	NE	NE	TOL	SY
5	I114 > V	BE	DE	NE	DE	NE	NE	NE	NE	TOL	SY
6	S172 > T	BE	IN	NE	DE	NE	NE	NE	NE	TOL	SY
7	L177 > S	PD	DE	NE	DE	NE	EFF	NE	NE	TOL	SY
8	G272 > R	PD	IN	DEL	DE	NE	EFF	NE	NE	TOL	NSY
9	L292 > Q	BE	DE	NE	DE	NE	NE	NE	NE	TOL	SY
10	E384 > Q	PD	IN	NE	DE	NE	EFF	NE	DIS	NT	NSY
***GDF1***											
1	S9 > G	UNK	DE	NE	DE	NE	NE	DEL	NE	NT	NSY
2	P254 > L	BE	DE	NE	DE	NE	NE	NE	NE	TOL	SY
***GDF2***											
1	R3 > C	PD	DE	NE	DE	NE	NE	NE	NE	TOL	SY
2	C14 > S	BE	DE	NE	DE	NE	NE	NE	NE	TOL	SY
3	G39 > R	BE	DE	NE	DE	NE	NE	NE	NE	TOL	SY
4	I215 > V	BE	DE	NE	DE	NE	NE	NE	NE	TOL	SY
5	G277 > S	BE	DE	NE	DE	NE	NE	NE	NE	TOL	SY
6	S308 > N	BE	DE	NE	IN	NE	NE	NE	NE	TOL	SY
7	T310 > M	BE	DE	NE	DE	NE	NE	NE	NE	TOL	SY
8	T323 > A	BE	DE	NE	DE	NE	NE	NE	NE	TOL	SY
9	G324 > A	BE	DE	NE	DE	NE	NE	NE	NE	NT	SY
***GDF3***											
1	E60 > A	BE	DE	NE	DE	NE	NE	NE	NE	TOL	SY
2	A101 > T	PD	DE	NE	DE	NE	NE	NE	NE	TOL	SY
3	I156 > T	BE	DE	NE	DE	NE	NE	NE	NE	TOL	SY
4	L211 > S	BE	DE	NE	DE	NE	EFF	NE	NE	TOL	SY
***GDF5***											
1	P87 > S	BE	DE	NE	DE	NE	NE	NE	NE	NT	SY
2	A214 > T	BE	IN	NE	DE	NE	NE	NE	NE	TOL	SY
***GDF6***											
1	E253 > K	BE	DE	NE	DE	NE	NE	NE	NE	TOL	SY
2	P257 > L	BE	IN	NE	IN	NE	NE	NE	NE	TOL	SY
3	G319 > R	PD	DE	NE	IN	NE	EFF	NE	NE	NT	SY
***GDF7***											
1	T98 > A	BE	DE	NE	DE	NE	NE	NE	NE	TOL	SY
2	V108 > A	BE	DE	NE	DE	NE	NE	NE	NE	TOL	SY
3	Q137 > E	BE	IN	NE	IN	NE	NE	NE	NE	TOL	SY
4	S190 > R	BE	IN	NE	IN	NE	NE	NE	NE	TOL	SY
5	S235 > R	BE	IN	NE	DE	NE	NE	NE	NE	TOL	SY
6	R304 > G	BE	DE	NE	DE	NE	NE	NE	NE	TOL	SY
***GDF9***											
1	K6 > N	PD	DE	NE	IN	NE	NE	NE	NE	TOL	SY
2	L49 > F	PD	DE	NE	DE	NE	NE	DEL	DIS	NT	NSY
3	N67 > K	BE	IN	NE	DE	NE	NE	NE	NE	TOL	SY
4	P77 > S	PD	DE	DEL	DE	NE	NE	DEL	DIS e	TOL	NSY
5	R84 > K	PD	DE	NE	DE	NE	NE	NE	NE	TOL	SY
6	E184 > A	BE	DE	NE	DE	NE	NE	NE	NE	TOL	SY
7	L260 > V	BE	DE	NE	DE	NE	NE	NE	NE	TOL	SY
8	D291 > G	BE	DE	NE	DE	NE	NE	NE	NE	TOL	SY
9	M402 > Q	BE	DE	NE	DE	NE	NE	NE	NE	TOL	SY
***GDF10***											
1	P42 > A	BE	DE	NE	DE	NE	NE	NE	NE	TOL	SY
2	T130 > M	BE	IN	NE	IN	NE	EFF	NE	DIS	NT	SY
3	P142 > H	PD	DE	NE	DE	NE	NE	NE	DIS	TOL	SY
4	P163 > S	BE	DE	NE	DE	NE	NE	NE	NE	TOL	SY
5	T180 > N	BE	IN	NE	DE	NE	NE	NE	NE	TOL	SY
6	S221 > A	PD	DE	NE	DE	NE	NE	NE	NE	TOL	SY
7	Q311 > H	BE	DE	NE	DE	NE	NE	NE	NE	TOL	SY
***GDF11***											
1	A40 > G	PD	DE	NE	DE	DIS	NE	DEL	NE	TOL	NSY
2	G41 > A	PD	IN	NE	IN	NE	NE	NE	NE	TOL	SY
***GDF15***											
1	P80 > S	BE	DE	NE	DE	NE	NE	NE	NE	TOL	SY
2	S135 > R	BE	IN	NE	IN	NE	NE	NE	NE	TOL	SY
3	A175 > S	BE	DE	NE	DE	NE	NE	NE	NE	TOL	SY
***AMH***											
1	F29 > S	BE	DE	NE	DE	NE	NE	NE	NE	TOL	SY
2	L34 > S	BE	DE	NE	DE	NE	NE	NE	NE	NT	SY
3	A50 > D	BE	IN	NE	DE	NE	NE	NE	NE	TOL	SY
4	S56 > P	BE	IN	NE	IN	NE	NE	NE	NE	TOL	SY
5	V89 > A	BE	DE	NE	DE	NE	NE	NE	NE	TOL	SY
6	A115 > S	BE	DE	NE	DE	NE	NE	NE	NE	NT	SY
7	N121 > D	BE	IN	NE	DE	NE	NE	NE	NE	TOL	SY
8	G122 > R	BE	IN	NE	DE	NE	NE	NE	NE	TOL	SY
9	P127 > A	BE	DE	NE	DE	NE	NE	NE	NE	NT	SY
10	V180 > L	BE	DE	NE	DE	NE	NE	NE	NE	TOL	SY
11	H216 > R	BE	IN	NE	DE	NE	NE	NE	NE	TOL	SY
12	S271 > P	BE	IN	NE	IN	NE	NE	NE	NE	TOL	SY
13	A273 > T	BE	DE	NE	DE	NE	NE	NE	NE	TOL	SY
14	A317 > R	BE	DE	NE	DE	NE	NE	NE	NE	TOL	SY
15	A334 > T	PD	DE	NE	DE	NE	NE	DEL	NE	NT	NSY
16	S432 > G	UNK	DE	NE	DE	NE	NE	NE	NE	TOL	SY
17	A468 > T	UNK	DE	NE	DE	NE	NE	NE	NE	TOL	SY
18	T534 > A	UNK	DE	NE	DE	NE	NE	NE	NE	TOL	SY
***LEFTY2***											
1	Q2 > R	BE	DE	NE	IN	NE	NE	NE	NE	TOL	SY
2	V12 > A	BE	DE	NE	DE	NE	NE	NE	NE	TOL	SY
3	T23 > M	PD	IN	NE	IN	NE	NE	DEL	NE	NT	SY
4	R26 > W	BE	DE	NE	DE	NE	EFF	NE	NE	NT	SY
5	D44 > N	BE	DE	NE	DE	NE	EFF	NE	NE	TOL	SY
6	A59 > T	BE	DE	NE	DE	NE	NE	NE	NE	TOL	SY
7	G70 > A	BE	IN	NE	DE	NE	EFF	NE	NE	TOL	SY
8	T91 > E	BE	IN	NE	DE	NE	NE	NE	NE	NT	SY
9	H97 > Y	PD	IN	NE	IN	NE	EFF	NE	NE	TOL	SY
10	T169 > S	BE	DE	NE	DE	NE	NE	NE	NE	TOL	SY
11	W230 > R	BE	DE	NE	DE	NE	EFF	NE	NE	TOL	SY
12	E260 > K	BE	IN	NE	DE	NE	EFF	NE	NE	NT	SY
13	A322 > T	BE	DE	NE	DE	NE	NE	NE	NE	TOL	SY
14	Q336 > R	BE	DE	NE	DE	NE	NE	NE	NE	TOL	SY
15	W356 > S	BE	DE	NE	DE	NE	EFF	NE	NE	TOL	SY
16	V359 > A	BE	DE	NE	DE	NE	NE	NE	NE	TOL	SY

BE: benign, PD: possibly damaging, DE: decrease, IN: increase, NE: neutral, DEL: deleterious, DI: disease, EFF: effect, UNK: unknown, NT: not tolerated, TOL: tolerated, SY: synonymous, NSY: non-synonymous.

## Data Availability

Not applicable.

## Supplementary Materials

The following supporting information can be downloaded at: https://www.mdpi.com/article/10.3390/genes13081302/s1, Figure S1: Molecular phylogenetic analysis of all the buffalo *TGF-β* genes for putative duplication detection; Figure S2: Comparative amino acid analysis of buffalo and cattle *TGFB2* gene; Figure S3: Comparative amino acid analysis of buffalo and cattle *TGFB3* gene; Figure S4: Comparative amino acid analysis of buffalo and cattle *INHBA* gene; Figure S5: Comparative amino acid analysis of buffalo and cattle *BMP7* gene; Figure S6: Comparative amino acid analysis of buffalo and cattle *INHBE* gene; Figure S7: Comparative amino acid analysis of buffalo and cattle *GDF6* gene; Figure S8: Comparative amino acid analysis of buffalo and cattle *BMP6* gene; Figure S9: Comparative amino acid analysis of buffalo and cattle *TGFB1* gene; Figure S10: Comparative amino acid analysis of buffalo and cattle *GDF11* gene; Figure S11: Comparative amino acid analysis of buffalo and cattle *BMP2* gene; Figure S12: Comparative amino acid analysis of buffalo and cattle *BMP4* gene; Figure S13: Comparative amino acid analysis of buffalo and cattle *BMP10* gene; Figure S14: Comparative amino acid analysis of buffalo and cattle *INHBB* gene; Figure S15: Comparative amino acid analysis of buffalo and cattle *BMP5* gene; Figure S16: Comparative amino acid analysis of buffalo and cattle *GDF1* gene; Figure S17: Comparative amino acid analysis of buffalo and cattle *GDF5* gene; Figure S18: Comparative amino acid analysis of buffalo and cattle *GDF15* gene; Figure S19: Comparative amino acid analysis of buffalo and cattle *MSTN* gene; Figure S20: Comparative amino acid analysis of buffalo and cattle *BMP8B* gene; Figure S21: Comparative amino acid analysis of buffalo and cattle *GDF3* gene; Figure S22: Comparative amino acid analysis of buffalo and cattle *GDF7* gene; Figure S23: Comparative amino acid analysis of buffalo and cattle *INHBC* gene; Figure S24: Comparative amino acid analysis of buffalo and cattle *NODAL* gene; Figure S25: Comparative amino acid analysis of buffalo and cattle *GDF10* gene; Figure S26: Comparative amino acid analysis of buffalo and cattle *BMP3* gene; Figure S27: Comparative amino acid analysis of buffalo and cattle *INHA* gene; Figure S28: Comparative amino acid analysis of buffalo and cattle *GDF2* gene; Figure S29: Comparative amino acid analysis of buffalo and cattle *GDF9* gene; Figure S30: Comparative amino acid analysis of buffalo and cattle *BMP8A* gene; Figure S31: Comparative amino acid analysis of buffalo and cattle *BMP15* gene; Figure S32: Comparative amino acid analysis of buffalo and cattle *LEFTY2* gene; Figure S33: Comparative amino acid analysis of buffalo and cattle *AMH* gene.
