# Supplementary material for: Molecular Characterization of TGF-Beta Gene Family in Buffalo to Identify Gene Duplication and Functional Mutations
Source: Genes (Basel). 2022 Jul 22;13(8):1302. doi: 10.3390/genes13081302 (PMC9331672; doi:10.3390/genes13081302)
Supplement: Supplementary file 1 [file genes-13-01302-s001.zip › Supplementary file.pdf]

Supplementary material

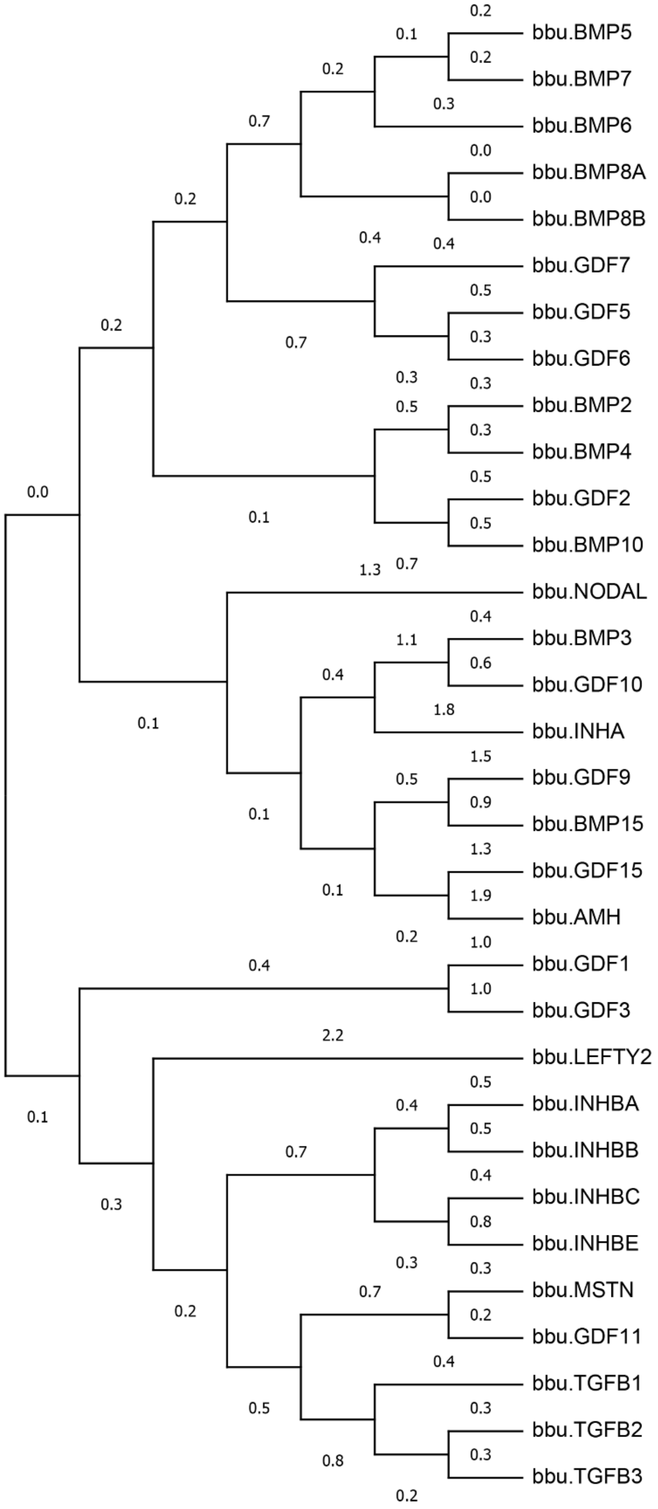

Figure S1. Molecular phylogenetic analysis of all the buffalo *TGF-β* genes for putative duplication detection

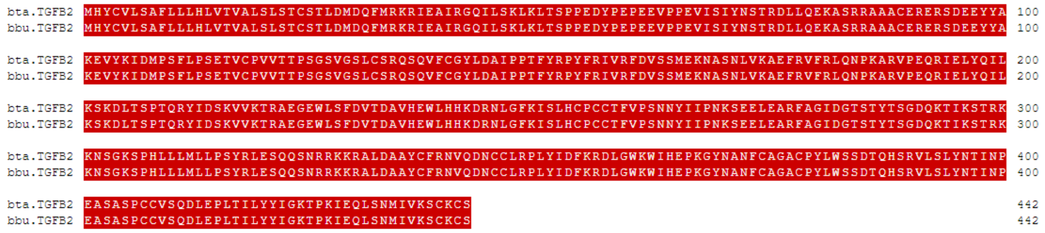

Figure S2. Comparative amino acid analysis of buffalo and cattle *TGFB2* gene

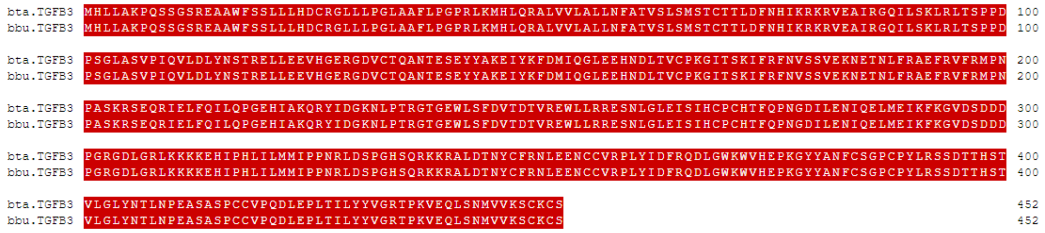

Figure S3. Comparative amino acid analysis of buffalo and cattle *TGFB3* gene

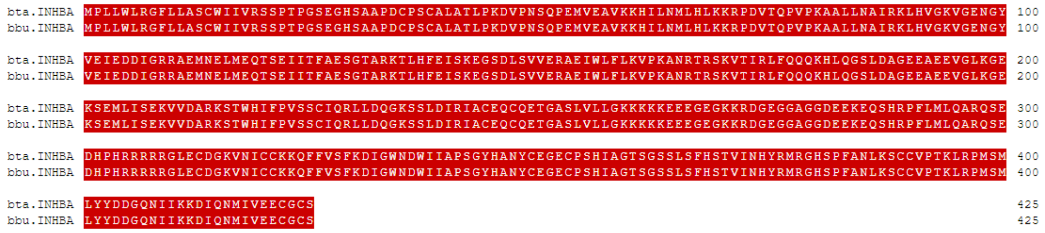

Figure S4. Comparative amino acid analysis of buffalo and cattle *INHBA* gene

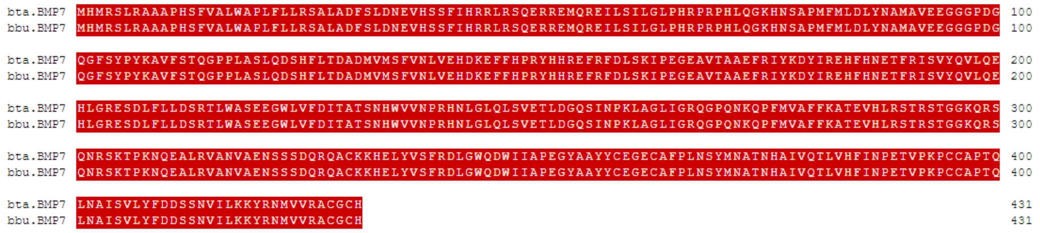

Figure S5. Comparative amino acid analysis of buffalo and cattle *BMP7* gene

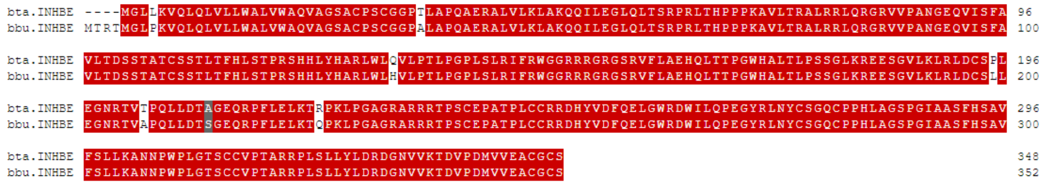

Figure S6. Comparative amino acid analysis of buffalo and cattle *INHBE* gene

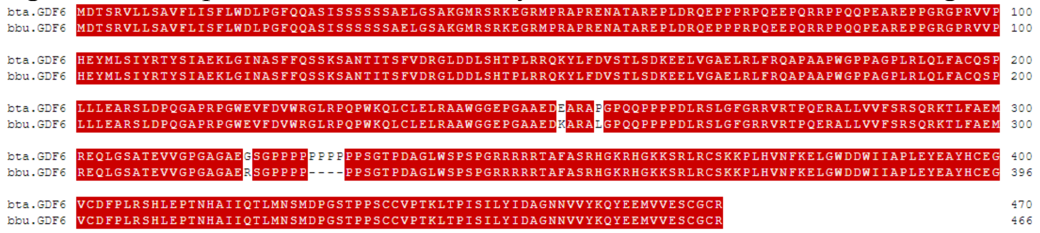

Figure S7. Comparative amino acid analysis of buffalo and cattle *GDF6* gene

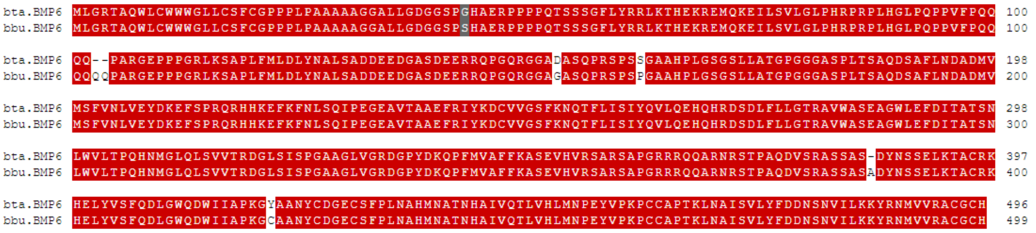

Figure S8. Comparative amino acid analysis of buffalo and cattle *BMP6* gene

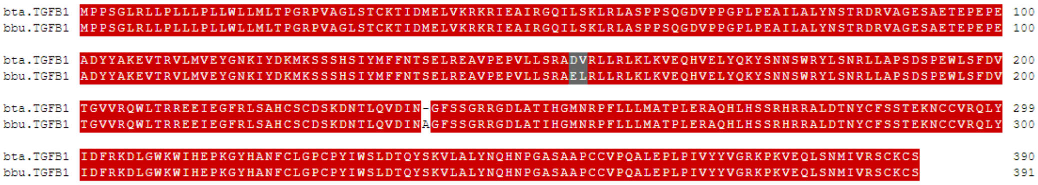

Figure S9. Comparative amino acid analysis of buffalo and cattle *TGFB1* gene

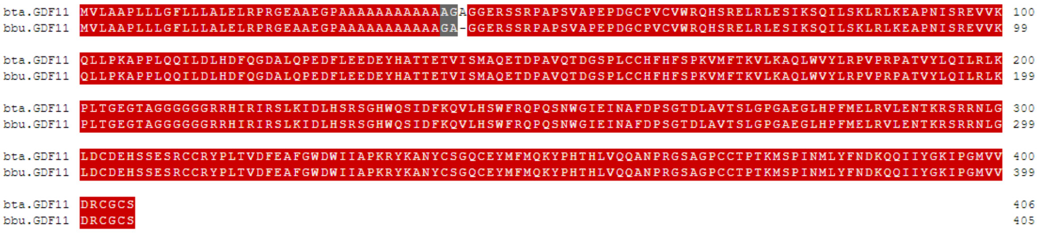

Figure S10. Comparative amino acid analysis of buffalo and cattle *GDF11* gene

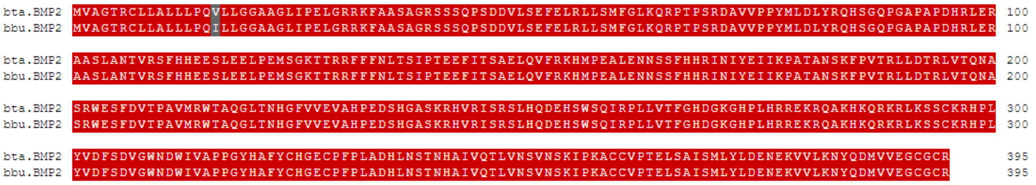

Figure S11. Comparative amino acid analysis of buffalo and cattle *BMP2* gene

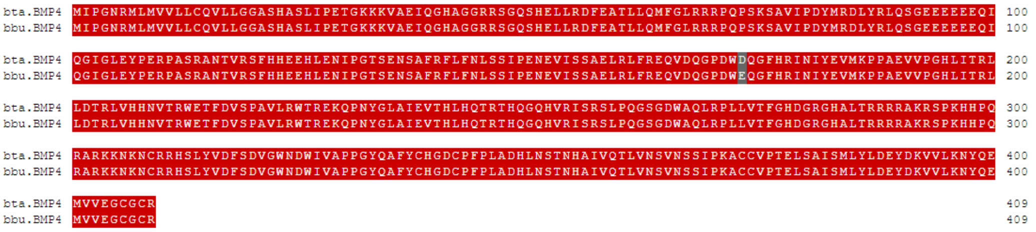

Figure S12. Comparative amino acid analysis of buffalo and cattle *BMP4* gene

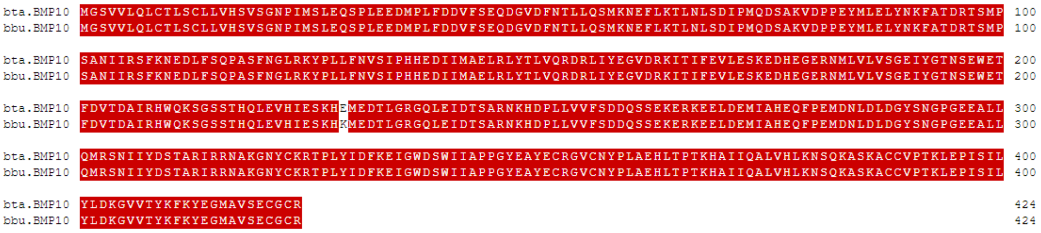

Figure S13. Comparative amino acid analysis of buffalo and cattle *BMP10* gene

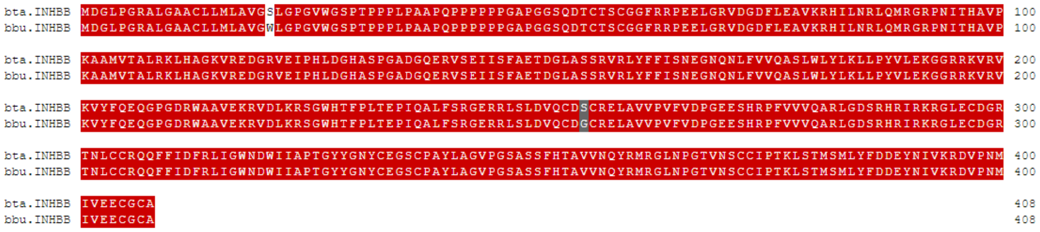

Figure S14. Comparative amino acid analysis of buffalo and cattle *INHBB* gene

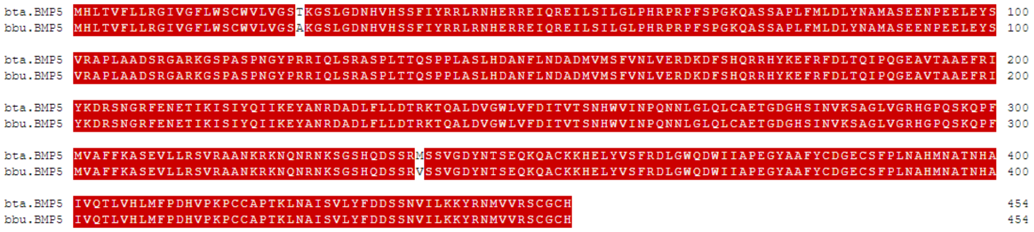

Figure S15. Comparative amino acid analysis of buffalo and cattle *BMP5* gene

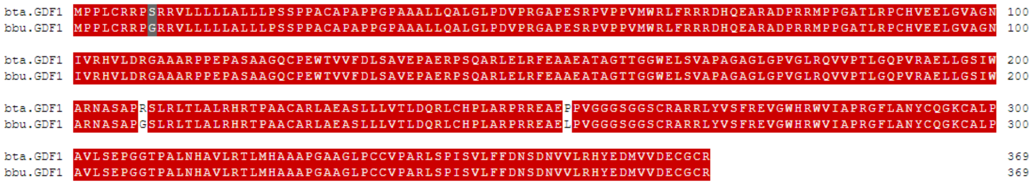

Figure S16. Comparative amino acid analysis of buffalo and cattle *GDF1* gene

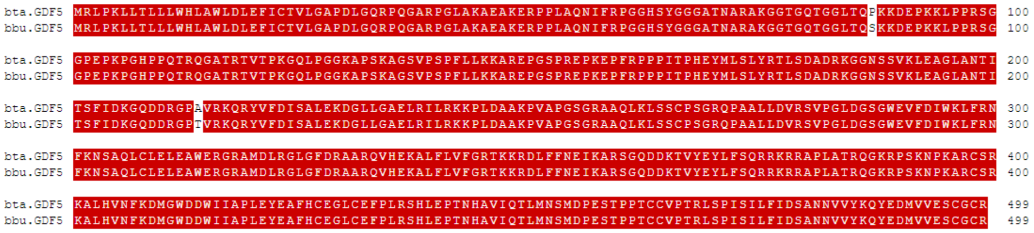

Figure S17. Comparative amino acid analysis of buffalo and cattle *GDF5* gene

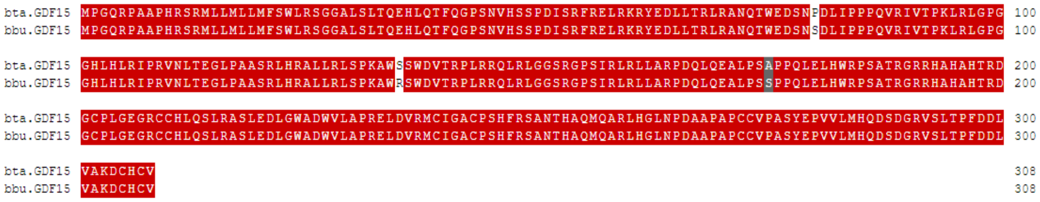

Figure S18. Comparative amino acid analysis of buffalo and cattle *GDF15* gene

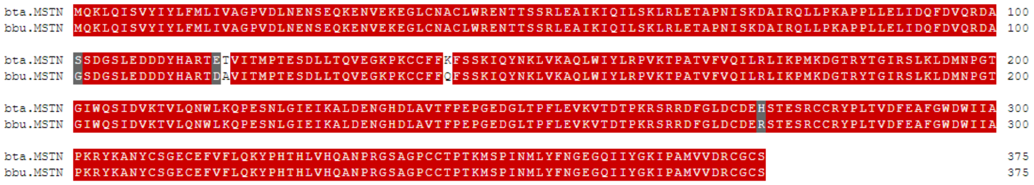

Figure S19. Comparative amino acid analysis of buffalo and cattle *MSTN* gene

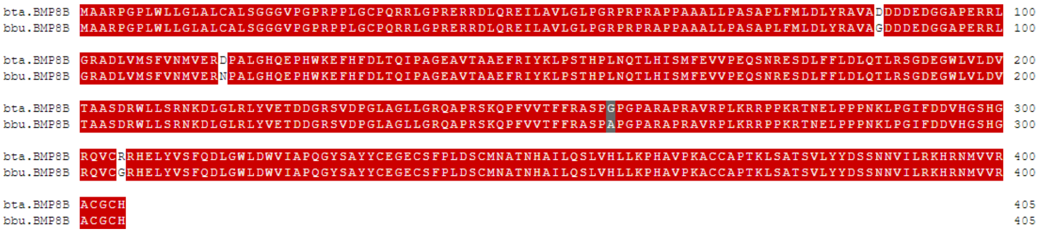

Figure S20. Comparative amino acid analysis of buffalo and cattle *BMP8B* gene

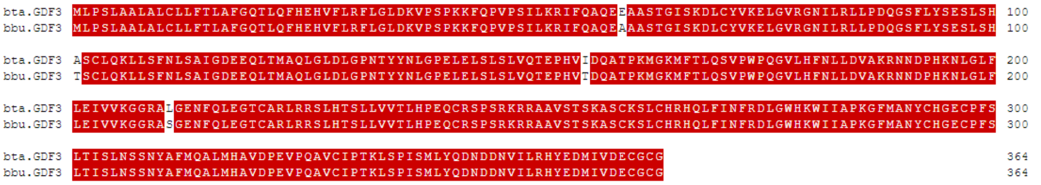

Figure S21. Comparative amino acid analysis of buffalo and cattle *GDF3* gene

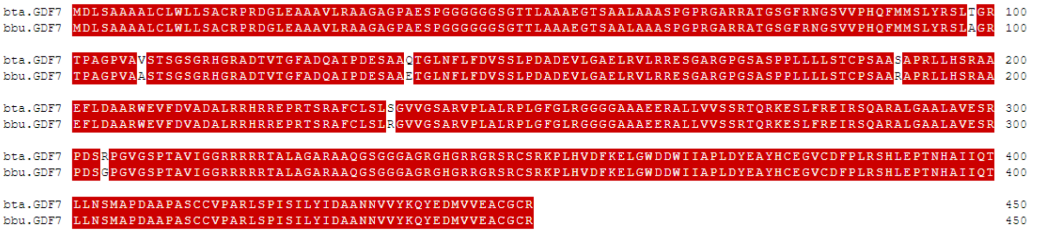

Figure S22. Comparative amino acid analysis of buffalo and cattle *GDF7* gene

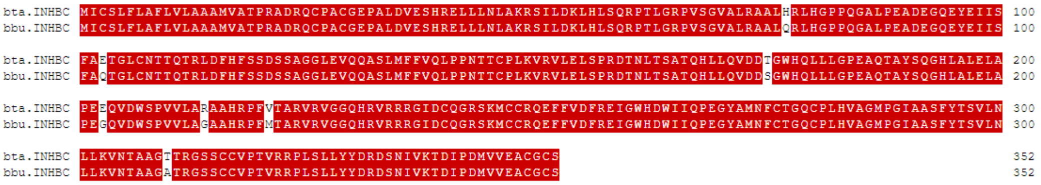

Figure S23. Comparative amino acid analysis of buffalo and cattle *INHBC* gene

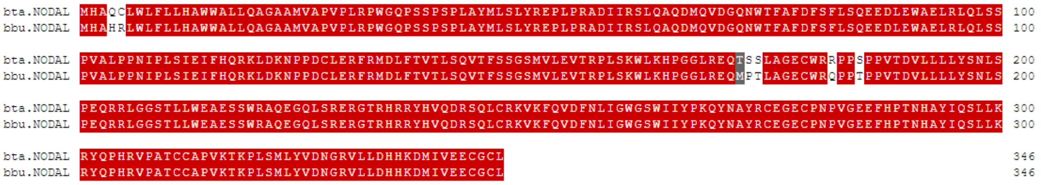

Figure S24. Comparative amino acid analysis of buffalo and cattle *NODAL* gene

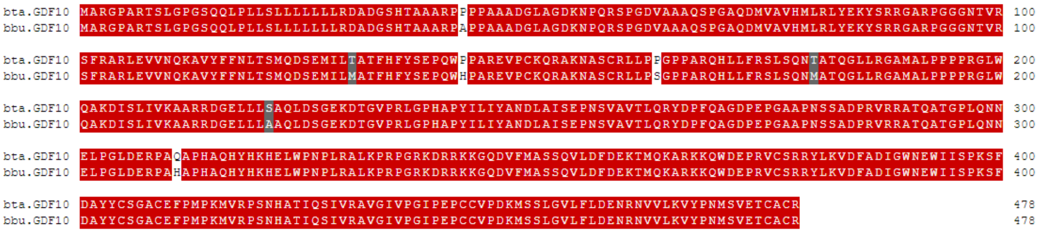

Figure S25. Comparative amino acid analysis of buffalo and cattle *GDF10* gene

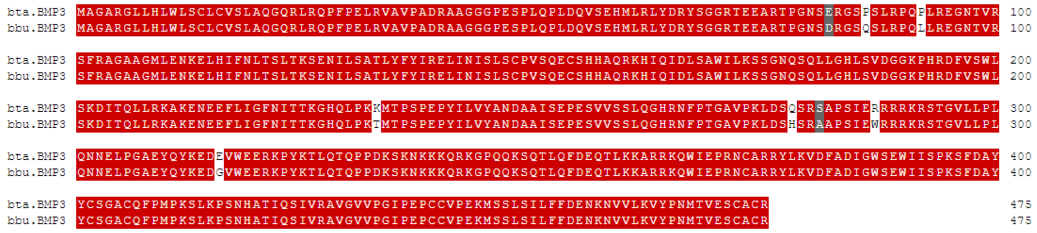

Figure S26. Comparative amino acid analysis of buffalo and cattle *BMP3* gene

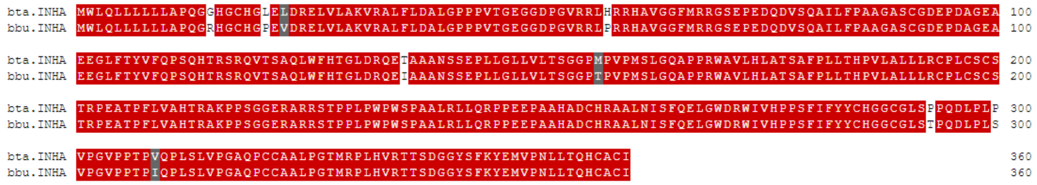

Figure S27. Comparative amino acid analysis of buffalo and cattle *INHA* gene

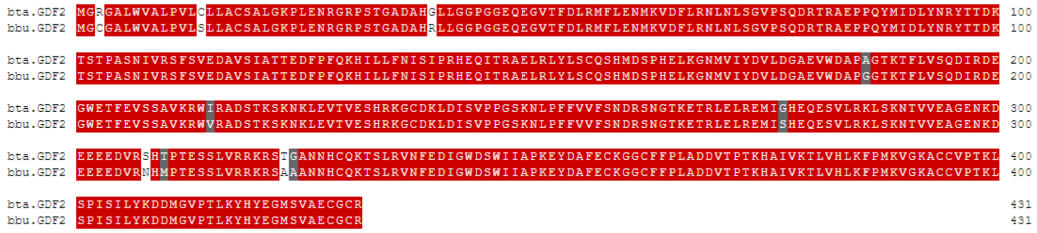

Figure S28. Comparative amino acid analysis of buffalo and cattle *GDF2* gene

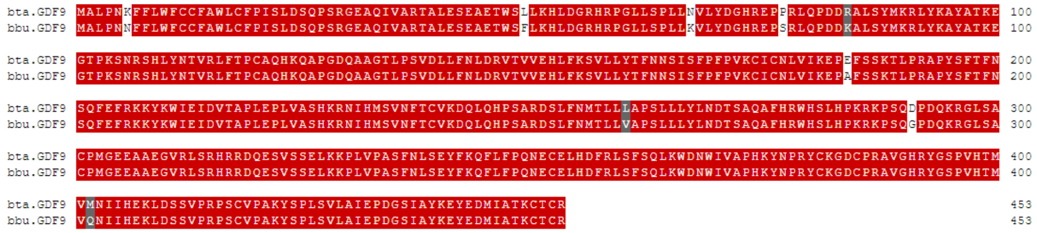

Figure S29. Comparative amino acid analysis of buffalo and cattle *GDF9* gene

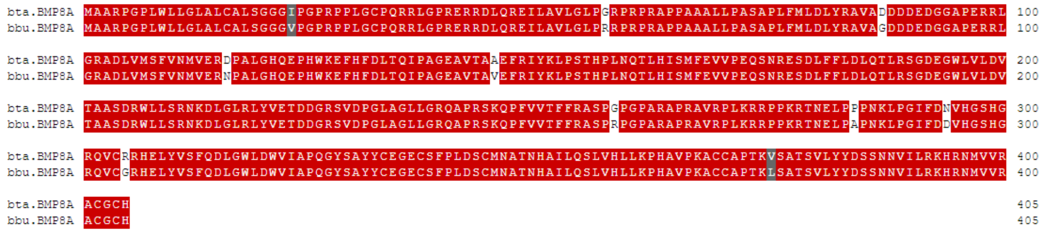

Figure S30. Comparative amino acid analysis of buffalo and cattle *BMP8A* gene

```
bta.BMP15  MVLLSILRILLLWGLVLFMEHRVQMTQVGQPSIAHLPEAPTLPLIQELLEEAPGKQQRKPRILGHPLRYMLELYQRSADASGHPRENRTIGATMVRLVRP  100
bbu.BMP15  MVLLSILRILLLWGLALFMEHRVQMTQVGQPSIAHLPEAPTLPLIQELLEEAPGKLQRKPRVLGHPLRYMLELYHRSADASGHPRENRTIGATMVRLVRP  100

bta.BMP15  LASVARPLRGSWHIQTLDFPLRPNRVAYQLVRATVVYRHQLHLTHSHLSCHVEPWVQKSPTNHFPSSGRGSSKPSLLPKAWTEMDIMEHVGQKLWNHKGR  200
bbu.BMP15  LASVARPLRGSWHVQTLDFPLRPNRVAYQLVRATVVYRHQLHLTHSHLSCHVEPWVQKSPTNHFPSSGRGSTKPSLSPKAWTEMDIMEHVGQKLWNHKGR  200

bta.BMP15  RVLRLRFVCQQPRGSEVREFWWHGTSSLDTVFLLLYFNDTQSVQKTKPLPKGLKEFTEKDPSLLLRRARQAGSIASEVPGPSREHDGPESNLCSLHPFQV  300
bbu.BMP15  RVLRLRFVCQQPRGSEVREFWWHGTSSLDTVFLLLYFNDTQSVQKTKPLPKGLKEFTEKDPSLLLRRARQARSIASEVPGPSREHDGPESNQCSLHPFQV  300

bta.BMP15  SFQQLGWDHWIIAPHLYTPNYCKGVCPRVLHYGLNSPNHAIIQNLVNELVDQSVPQPSCVPYKYVPISILLIEANGSILYKEYEGMIAQSCTCR  394
bbu.BMP15  SFQQLGWDHWIIAPHLYTPNYCKGVCPRVLHYGLNSPNHAIIQNLVNELVDQSVPQPSCVPYKYVPISILLIEANGSILYKEYQGMIAQSCTCR  394
```

Figure S31. Comparative amino acid analysis of buffalo and cattle *BMP15* gene

```
bta.LEFTY2  MQPLWLCWALWVLPLAGPGAALTEERILDSLLQQLHLSEVPIVDKATVEGLVIPAHVRAQYVALLQRGHGARSRGKRFSQNFREVVGRFLTSEASSHLLV  100
bbu.LEFTY2  MRPLWLCWALWALPLAGPGAALMEEWILDSLLQQLHLSEVPIVNKATVEGLVIPAHVRTQYVALLQRGHAARSRGKRFSQNFREVVGRFLESEASSYLLV  100

bta.LEFTY2  FDMEQRLPPRSELVQAVLRLFQEPVPRAALRRHERLFPRSDRARVTVQWLHVRDDGSNRTALIDSRLVTIHESGWKALDVTEAVNFWQQLRSPRQPLLLQ  200
bbu.LEFTY2  FDMEQRLPPRSELVQAVLRLFQEPVPRAALRRHERLFPRSDRARVTVQWLHVRDDGSNRTALIDSRLVSIHESGWKALDVTEAVNFWQQLRSPRQPLLLQ  200

bta.LEFTY2  VLVQREHLGPLASSAHRLVRFAPQGPSSGWQGEPQLELHTLDLRDYGAQGNCDPKAPVTEGTRCCRQEMYIDLQGMKWAENWVLEPPGFLAYECVGTCQQ  300
bbu.LEFTY2  VLVQREHLGPLASSAHRLVRFAPQGPSSGRQGEPQLELHTLDLRDYGAQGNCDPKAPVTKGTRCCRQEMYIDLQGMKWAENWVLEPPGFLAYECVGTCQQ  300

bta.LEFTY2  PPESLTFKWPFLGPRQCIASEATSLPMIVSIQEGGQLQPQVVSLPNMRVQTCSCAWDGVLVPRKLEP  367
bbu.LEFTY2  PPESLTFKWPFLGPRQCIASETTSLPMIVSIQEGGRLQPQVVSLPNMRVQTCSCASDGALVPRKLEP  367
```

Figure S32. Comparative amino acid analysis of buffalo and cattle *LEFTY2* gene

```
bta.AMH  MPGPSLSLALVLSAMGALLRPGTPREEVFSTSALPREQATGSGALIFQQAWDWPLSSLWLPGSPLDPLCLVTLHGSGNGSRAPLRVVGVLSSYEQAFLEA  100
bbu.AMH  MPGPSLSLALVLSAMGALLRPGTPREEVSSTSASPREQATGSGALIFQQDWDWPLPSLWLPGSPLDPLCLVTLHGSGNGSRAPLRVVGALSSYEQAFLEA  100

bta.AMH  VRRTHWGLSDLTTFAVCPAGNGQPVLPHLQRLQAWLGEPGGRWLVVLHLEEVTWEPTPLLRFQEPPPGGASPPELALLVVYPGPGLEVTVTGAGLPGTQS  200
bbu.AMH  VRRTHWGLSDLTTFSVCPAGDRQPVLAHLQRLQAWLGEPGGRWLVVLHLEEVTWEPTPLLRFQEPPPGGASPPELALLVLYPGPGLEVTVTGAGLPGTQS  200

bta.AMH  LCLTADSDFLALVVDHPEGAWRRPGLALTLRRRGNGALLSTAQLQALLFGADSRCFTRKTPALLLLLPARSSAPMPAHGRLDLVPFPQPRASPEPEEAPP  300
bbu.AMH  LCLTADSDFLALVVDRPEGAWRRPGLALTLRRRGNGALLSTAQLQALLFGADSRCFTRKTPALLLLLPARPSTPMPAHGRLDLVPFPQPRASPEPEEAPP  300

bta.AMH  SADPFLETLTRLVRALAGPPARASPPRLALDPGALAGFPQGQVNLSDPAALERLLDGEEPLLLLLPPTAATTGVPATPQGPKSPLWAAGLARRVAAELQA  400
bbu.AMH  SADPFLETLTRLVRALRGPPARASPPRLALDPGTLAGFPQGQVNLSDPAALERLLDGEEPLLLLLPPTAATTGVPATPQGPKSPLWAAGLARRVAAELQA  400

bta.AMH  VAAELRALPGLPPAAPPLLARLLALCPGNPDSPGGPLRALLLLKALQGLRAEWRGRERSGSARAQRSAGAAAADGPCALRELSVDLRAERSVLIPETYQA  500
bbu.AMH  VAAELRALPGLPPAAPPLLARLLALCPGNPDGPGGPLRALLLLKALQGLRAEWRGRERSGSARAQRSTGAAAADGPCALRELSVDLRAERSVLIPETYQA  500

bta.AMH  NNCQGACGWPQSDRNPRYGNHVVLLLKMQARGATLARPPCCVPTAYTGKLLISLSEERISAHHVPNMVATECGCR  575
bbu.AMH  NNCQGACGWPQSDRNPRYGNHVVLLLKMQARGAALARPPCCVPTAYTGKLLISLSEERISAHHVPNMVATECGCR  575
```

Figure S33. Comparative amino acid analysis of buffalo and cattle *AMH* gene
